# Effect of microbial inoculants on fermentation quality and aerobic stability of sweet potato vine silage

**DOI:** 10.5713/ajas.18.0264

**Published:** 2018-07-26

**Authors:** Young Ho Joo, Dong Hyeon Kim, Dimas H. V. Paradhipta, Hyuk Jun Lee, Sardar M. Amanullah, Sang Bum Kim, Jong Soo Chang, Sam Churl Kim

**Affiliations:** 1 Division of Applied Life Science (BK21Plus, Insti. of Agri. & Life Sci.), Gyeongsang National University, Jinju 52828, Korea; 2 Biotechnology Division, Bangladesh Livestock Research Institute, Savar, Dhaka-1341, Bangladesh; 3 Dairy Science Division, National Institute of Animal Science, RDA, Cheonan 31000, Korea; 4 Department of Agricultural Science, Korea National Open University, Seoul 03087, Korea

**Keywords:** Aerobic Stability, Microbial Inoculant, Silage, Sweet Potato Vine

## Abstract

**Objective:**

This study was conducted to evaluate the effect of homo or hetero fermentative inoculants on fermentation quality and aerobic stability of sweet potato vine (SPV) silage containing Italian ryegrass hay as moisture absorbent.

**Methods:**

The SPV was harvested at 15% dry matter, mixed with Italian ryegrass hay at 1:1 ratio on a fresh weight basis, and chopped to 3 to 5 cm length. After then, the chopped forage mixture was ensiled into 20-L mini silos in quadruplicate for 7, 48, and 100 days after application of microbial inoculants at 1.2×10^5^ colony forming units (cfu)/g of forage following: no inoculant (CON), *Lactobacillus plantarum* as a homo fermentative (LP), *Lactobacillus buchneri* as a hetero fermentative (LB), and mixture of LP and LB at 1:1 ratio as a combo fermentative (MIX).

**Results:**

The LP and MIX silages had lowest pH (p<0.001) on 7 and 48 days, while MIX and CON silages had greatest lactate concentrations (p<0.05) on 7 and 48 days, respectively. Acetate concentrations were highest (p<0.01) in LB and MIX silages on 7 days, and in LB silage on 48 days, while lactate to acetate ratios were lowest (p<0.001) in LB silages. The chemical compositions and nutrient digestibility of silage ensiled for 100 days was not affected by inoculants. On 100 days of ensiling, LB silage had lowest (p<0.01) lactate concentration and lactate to acetate ratio, but highest acetate concentration. Aerobic stability was highest (p<0.001) in LB silage followed in MIX silage. On contrast, LB silage had lowest (p<0.05) lactic acid bacteria and mold.

**Conclusion:**

The results indicated that application of LB solely had a better effect on aerobic stability than not only LP, but also MIX. However, LP application did not show beneficial effects from the viewpoints of fermentation quality and aerobic stability compared to CON.

## INTRODUCTION

Sweet potato is one of the most important food crops, which is cultivated widely in tropical and subtropical regions of the world, particularly in Asia (78%) and Africa (18%). Sweet potato vine (SPV) consisting leaf, stem, and stalk is the by-product of its root, and considered as a good feedstuff for ruminants due to the high concentrations of crude protein (CP) and water soluble carbohydrate (WSC) [1,2]. The SPV contains approximately 12.3% dry matter (DM), 13.5% CP, 50.6% neutral detergent fiber (NDF), 33.9% acid detergent fiber (ADF) [1], and 46.8% WSC [2]. Based on these nutritive values, SPV provides a nutritional requirement that acceptable for growth in heifers [1]. Also, sweet potato leaf could be an alternative as a cheap nitrogen source in the diets for goats and pigs [3]. However, the utilization of SPV for ruminant is limited due to the rapid spoilage by the growth of undesirable microbes, which might be caused by high WSC and moisture contents of SPV. Application of SPV as silage can be chosen to improve its preservation quality of SPV [2,4]. The ensiled SPV had shown the beneficial effects on growing pigs and goats [5–7]. However, SPV was spoiled within 5 h of wilting period in our preliminary study (Data unpublished) while SPV was wilted to reach the ideal moisture content (60% to 70%) for making silage. With these cautions, addition of the moisture absorbent might be recommended to reduce the moisture content of SPV.

Application of bacterial inoculant has been used to improve the fermentation quality and aerobic stability of silages [8]. In general, silage fermentation is influenced by the role of lactic acid bacteria (LAB) during ensiling. A previous study reported that silage inoculated with a homo fermentative LAB enhanced the fermentation quality [9], while a hetero fermentative LAB improved the aerobic stability [10]. The combination of homo and hetero fermentative LAB improved not only fermentation quality, but also aerobic stability [11]. However, inoculants effect on silage varies by species, maturity, and chemical composition of forages. The aerobic stability of SPV silage might be a critical factor to ensure the quality of silage with a high WSC content. Due to its high WSC concentration, yeast and mold will rapidly spoil the SPV silage after silo open. However, studies on aerobic stability of SPV silage are limited.

In this study, the SPV was mixed with a moisture absorbent instead of a wilting process, and then, ensiled with single or combo inoculants. *Lactobacillus plantarum* (*L. plantarum*) as a homo fermentative LAB was expected to drop down the pH rapidly by high production of lactate, while *Lactobacillus buchneri* (*L. buchneri*) as a hetero fermentative LAB would decrease pH slowly but would produce more acetate to enhance aerobic stability. Therefore, this study was designed to determine the effect of homo, hetero, or combo inoculants on fermentation quality and aerobic stability of SPV silage.

## MATERIALS AND METHODS

### Silage production

Sweet potato was grown at the commercial sweet potato farm, Heanam, Korea, and the SPV was collected just before the sweet potato harvesting at 15% DM. The harvested SPV mixed at 1:1 ratio with Italian ryegrass (IRG) hay as a moisture absorbent on a fresh weight basis. The mixed forage chopped to 3 to 5 cm length, filled 200 kg for each treatment and then, treated with inoculants as follows: i) no inoculant, applied 1% distilled water in fresh forage (CON); ii) commercial *L. plantarum* (CMbio, Anseong, Korea) as a homo fermentative LAB, applied in fresh forage at 1.5×10^5^ colony forming units (cfu)/g (LP); iii) *L. buchneri* KACC12416 (Korean Culture Center of Microorganism, Seoul, Korea) as a hetero fermentative LAB, applied in fresh forage at 1.2×10^5^ cfu/g (LB); and iv) Mixture of LP and LB at 1:1 ratio as a combo fermentative (MIX). Previously, the microbial levels of LP and LB were counted by lactobacilli MRS agar media (MRS; Difco, Detroit, MI, USA) to calculate the recommended application rate. All the treatments were packed into 20 L silos for 0, 7, 48, and 100 days of the ensiling period with 4 replications.

### Laboratory analysis

Just before ensiling, the mixed forages were subsampled (1 kg) in quadruplicates for their chemical composition and *in vitro* digestibility. Each silo was opened on the assigned day and subsampled for silage extraction (20 g). The silage (2 kg) ensiled for 100 days was sub-sampled for chemical composition, *in vitro* digestibility, and aerobic stability. The sub-sampled forage and silage (500 g) were dried at 65°C for 48 h and ground to pass 1-mm screen using a cutting mill (SHINMYUNG ELECTRIC Co., Ltd, Gimpo, Korea) for the measurement of chemical composition and *in vitro* digestibility. The DM content was analyzed by dried in a forced-air oven at 105°C for 24 h. Crude ash was determined with a muffle furnace at 550°C for 5 h. The CP and ether extract were measured by the producers of Kjeldahl and the Soxhlet [12], respectively. The NDF and ADF were determined by using Ankom 200 fiber analyzer (Ankom Technology, Macedon, NY, USA) following Van Soest method [13]. Hemicellulose content was calculated by the difference between NDF and ADF. The *in vitro* DM digestibility (IVD_DM_) and *in vitro* NDF digestibility (IVD_NDF_) was determined by following Tilley and Terry protocol [14] using an Ankom Daisy (Ankom Technology, Macedon, NY, USA).

Twenty grams of silage was blended with 200 mL of sterile ultrapure water for 30 seconds and filtered through 2 layers of cheesecloth to make silage extraction. The silage extract was used to determine the pH, lactate, volatile fatty acid (VFA), ammonia-N, and microbial levels. The pH was measured by pH meter (SevenEasy, Mettler Toledo, Greifensee, Switzerland). Ammonia-N was determined using a colorimetric method [15]. The silage extraction was centrifuged at 5,645×*g* for 15 min and collected the supernatant for lactate and VFA analyses. The concentrations of lactate and VFA were determined using HPLC (L-2200, Hitachi, Tokyo, Japan) fitted with a UV detector (L-2400; Hitachi, Japan) and a column (Metacarb 87H; Varian, Palo Alto, CA, USA) [16]. Silage extract (first dilution) from 100 days of ensiled silage was continued in several dilutions (10^−5^ to 10^−7^) to determine microbial levels such as LAB, yeast and mold. The silage extract was plated in triplicate on selective agar medium with three replications for each dilution series. The lactobacilli MRS agar media was used for LAB level, and potato dextrose agar (PDA; Difco, USA) for yeast and mold levels. The MRS agar plates were placed in a CO_2_ incubator (Thermo Scientific, Waltham, MA, USA) at 39°C for 24 h, while PDA plates were incubated at 39°C for 72 h in an aerobic incubator (Johnsam Corp., Boocheon, Korea). The differentiation among yeast and mold in PDA culture was conducted based on the colony type. Visible colony was counted from the plates and the microbial level data was presented in log10 cfu per gram of silage.

Aerobic stability was measured by placing sample (1 kg) into polystyrene box in aerobic condition at room temperature (20°C). The temperature was recorded by a thermocouple, a sensor (MORGAN TR-60CH, Hong Kong, China) that placed at the geometric center of each sample. Data were collected every 30 min during 20 days. Aerobic stability was measured by the time (h) before a 2°C increase in silage temperature above the ambient temperature [17].

### Statistical analysis

Collected data were analyzed using analysis of variance by Statistical Analysis System 9.3 (SAS, Version 9. Cary, NC, USA) [18]. Mean separation was performed by Tukey test and the significant differences were declared at p<0.05.

## RESULTS

### Chemical composition and *in vitro* digestibility of fresh forage

The chemical composition and *in vitro* digestibility of fresh SPV mixed with IRG hay just before ensiling were not affected by bacterial inoculant applications (Table 1). The mean concentrations of DM, CP, NDF, ADF, IVD_DM_, and IVD_NDF_ of fresh forage across the treatments were 39.3%, 6.50%, 59.3%, 38.8%, 57.8%, and 38.8%, respectively.

### Fermentation indices on 7, 48, and 100 days

On 7 days of ensiling, all silages applied inoculant had lower pH (p<0.001; 4.13 vs 4.68) than CON silage, especially lowest in LP and MIX silages (Table 2). Lactate concentration of MIX silage was higher (p = 0.007; 2.40% vs 1.23% and 1.30%) than that of CON and LB silages, while acetate concentrations of LB and MIX silages were higher (p<0.001) than that of CON and LP silages. Lactate to acetate ratio (p<0.001; 5.56 vs 1.35) was highest in LP silage, but lowest in LB silage. On 48 days of ensiling, LB silage had highest pH (p<0.001; 4.40 vs 4.00 and 3.99), while LP and MIX silages had lowest. Lactate concentration in CON silage was highest (p = 0.030; 3.28% vs 1.41%), but lowest in LB silage. Acetate concentration of LB silage was highest (p = 0.007; 2.54% vs 0.58%), but lowest in LP silage. In contrast, lactate to acetate ratio was highest (p<0.001; 5.05 vs 0.56) in LP silage, but lowest in LB silage. Propionate and butyrate were not detected in all silages on 7 and 48 days. On 100 days of ensiling, pH and ammonia-N concentration were not affected by inoculant applications. However, lactate concentrations of LP and MIX silages were higher (p = 0.009; 2.73% and 2.82% vs 1.55%) than that of LB silage. In contrast, acetate concentration of LB silage was higher (p = 0.005; 1.66% vs 0.76%, 0.47%, and 0.81%) than that of the others. Lactate to acetate ratio (p = 0.001; 5.81 vs 0.93) was highest in LP silage, but lowest in LB silage. Propionate and butyrate were not detected in all silages on 100 days.

### Chemical composition and *in vitro* digestibility on 100 days

The bacterial inoculant applications were not affected on chemical compositions and *in vitro* digestibility of SPV silage ensiled for 100 days (Table 3). The mean concentrations of DM, CP, NDF, IVD_DM_, and IVD_NDF_ of SPV silage across the treatments were 38.0%, 7.79%, 62.1%, 58.7%, and 41.3%, respectively.

### Aerobic stability and microbial levels on 100 days

Aerobic stability was highest (p<0.001; 149.2 vs 39.9 and 36.4 h) in LB silage, but lowest in CON and LP silages (Table 4). The LAB was higher (p = 0.008; 6.60 vs 6.04 and 6.20 cfu/g) in MIX silage than in LP and LB silages. The yeast was not affected by inoculant (p = 0.085) in all silages, while mold was higher (p = 0.018; 5.50 vs 4.09 cfu/g) in LP silage than in LB silage.

## DISCUSSION

The IRG hay was added into SPV to reduce moisture content considering the obstacles in the wilting process. In the other side, a forage fermentation containing excess moisture will lead to the growth of clostridia [8]. Addition of IRG into SPV forage decreased moisture to ideal concentration (60.7%) but changed the chemical composition of SPV silage. After a wilting process for 24 h the original SPV silage contained 27.2% DM, 10.9% CP, 49.8% NDF, and 41.6% ADF [4], which has lower DM and NDF, and higher CP concentrations compared to our SPV silage. A change of chemical composition in our SPV silage might show the different effects on fermentation quality and aerobic stability compared to original SPV.

Inoculant applications influenced the fermentation indices of SPV silage on 7, 48, and 100 days of the ensiling period. On 7 days as early period of ensiling, all inoculants stimulated fermentation that resulted in pH less than 4.5, while CON showed the higher pH due to lower organic acid production (lactate plus acetate; 1.58% vs 1.97%, 2.26%, and 3.20%). The main purpose of applied inoculants is to stimulate the growth of LAB which can increase lactate production [8,11]. Without inoculant, the lactate production could occur at a slow rate. On 48 days of ensiling, the highest pH did not resulted in CON anymore but it was occurred in LB considering the lowest lactate to acetate ratio that indicated higher production of lactate than acetate. During this period, a hetero fermentative LAB converted lactate into acetate and other secondary products such as 1,2-propanediol and a trace amount of ethanol [19], which decreased the lactate proportion during ensiling. This could result in a declining pH rate during fermentation. This case has been reported by previous studies [8,20], where LB applied to silages produced a higher pH and a lower lactate concentration. In the present study, a higher proportion of lactate in CON, LP, and MIX silages decreased pH lower than LB silage on 48 days of ensiling. Both of LP and MIX provided a homo fermentative LAB resulting in a relatively low pH condition [8], while fermentation in CON silage could be led by homo fermentative LAB resulting in a relatively low pH condition [21]. On the other side, no detection of ammonia-N on 7 and 48 days of ensiling might be caused by rapid acidification in all silages (pH 3.96 to 4.68). Even though the ammonia-N was detected on 100 days of ensiling, it was very low (0.35% to 0.40%) and was not affected by inoculant applications. The concentration of ammonia-N in the silage reflects the protein degradation during ensiling [20]. The rapid acidification of silage in all treatments could inhibit the undesirable microbes such as mold and clostridia, which decreased proteolysis activity in the silage [8]. Also, this rapid acidification might be a reason of no butyric acid in any SPV silages, which indicated a good fermentation process in the present study. In the other organic acid production, silage treated with CON, LP, and MIX produced high lactate concentration considering homo lactic fermentation due to high WSC concentration, but it did not show the differences. On the other side, LB produced high acetate as the response of hetero-lactic fermentation in the present study. Inoculated silage with MIX did not show the dual effects in enhancing lactate and acetate as well as we expected, and this case was similar with a previous study [22].

The previous study reported that applied *Lactobacillus formosensis* in SPV silage reduced DM loss and increased *in vitro* rumen N digestibility [23]. Also, application of LAB as silage inoculants potentially increased the digestibility in rumen [24]. However, the inoculant applications did not show the effects on chemical compositions and *in vitro* digestibility after ensiled for 100 days in the present study. These disagreements might be due to the addition of IRG hay, which could result in the different effects on DM loss and *in vitro* rumen digestibility. Additionally, inoculant application does not always promise the improvement of silage quality. Previous studies had resulted in the various effects on chemical composition and *in vitro* digestibility of silage by inoculant applications, which were influenced by difference type of forage and LAB strains [11,17,22,25,26].

Aerobic stability is an important parameter in determining SPV silage quality considering its high WSC content. After ensiled, original SPV silage still contains a lot of WSC, approximately 13.9% of DM [23]. Silage treated with LB resulted in the highest aerobic stability, particularly LB silage was 4 times greater (149.2 vs 36.4 h) than LP silage in the present study. This result might be due to the highest acetate production in LB silage. It also had been reported that aerobic stability is more affected by the antifungal agents produced in ensiling such as acetate and propionate than yeast level [26,27]. This speculation also agrees with the results in the present study. Acetate has an antimicrobial effect that acts as an inhibitor of spoilage microorganism in aerobic conditions with the concentration of acetate increasing aerobic stability exponentially [28,29]. Previously, inoculation of *L. buchneri* in several kinds of silages also increased aerobic stability [11,25]. On the other side, CON and LP had lowest aerobic stability. The presence of lactate had a negative effect on aerobic stability because lactate can be used as an energy source for spoilage microorganisms [27]. Inoculation of silage with a homo fermentative LAB has been reported before, and it caused rapid spoilage when silage was exposed by the air [25,26]. Even though MIX provided a hetero fermentative LAB to stimulate acetate production, it has not been able to increase aerobic stability as well as LB. On the other side, LAB could control the growth of yeast and mold during fermentation by antifungal activity [30]. However, applied LAB as silage inoculant has shown the various results on the microbial levels of silages, which depend on fermentation indices during the ensiling [11,25,26]. Even though the rapid acidification had shown in LP compare to in LB in the present study (Table 2), inoculation of LB had less mold level. This might be due to the higher acetate concentration in LB than in LP, as acetate is known to have a stronger antimicrobial activity than lactate [28]. Based on the results of this study, application of LB in SPV silage had the greatest effect on aerobic stability compared to other inoculants. However, application of LP did not show any beneficial effects on fermentation quality and aerobic stability of SPV silage when compared to CON.

## Figures and Tables

**Table 1:** Effects of homo and hetero fermentative inoculants on chemical composition and *in vitro* digestibility of fresh sweet potato vine with Italian ryegrass hay (%, dry matter)

Item	Treatment^1)^				SEM	p value

	CON	LP	LB	MIX		
Dry matter	39.8	39.0	39.4	38.9	1.038	0.689
Crude protein	6.53	6.41	6.58	6.48	0.255	0.809
Ether extract	1.50	1.47	1.46	1.50	0.058	0.785
Crude ash	7.40	7.32	7.45	7.45	0.110	0.406
Neutral detergent fiber	59.1	58.6	59.6	60.0	1.157	0.400
Acid detergent fiber	39.1	38.4	38.3	39.4	0.666	0.111
Hemicellulose	20.0	20.2	21.3	20.6	0.940	0.771
IVD_DM_	58.0	58.1	57.6	57.6	1.189	0.398
IVD_NDF_	39.0	38.8	38.5	39.0	1.097	0.297

SEM, standard error of the mean; IVD_DM_, *in vitro* dry matter digestibility; IVD_NDF_, *in vitro* neutral detergent fiber digestibility.

CON, silage with no inoculant; LP, silage inoculated with *L. plantarum* at 1.2×10^5^ cfu/g of forage; LB, silage inoculated with *L. buchneri* at 1.2×10^5^ cfu/g of forage; MIX, silage inoculated with mixture of LP and LB at 1:1 ratio.

**Table 2:** Effects of homo and hetero fermentative inoculants on fermentation indices of sweet potato vine mixed with Italian ryegrass hay ensiled for 7, 48, and 100 days

Item	Treatment1)				SEM	p value

	CON	LP	LB	MIX		
Ensiled for 7 days						
pH	4.68^a^	4.02^c^	4.42^b^	3.96^c^	0.066	<0.001
Ammonia-N (%)	ND	ND	ND	ND	-	-
Lactate (%)	1.23^b^	1.67^ab^	1.30^b^	2.40^a^	0.819	0.007
Acetate (%)	0.35^b^	0.30^b^	0.96^a^	0.80^a^	0.131	<0.001
Lactate:acetate ratio	3.51^ab^	5.56^a^	1.35^c^	3.01^b^	0.183	<0.001
Ensiled for 48 days						
pH	4.12^b^	4.00^c^	4.40^a^	3.99^c^	0.034	<0.001
Ammonia-N (%)	ND	ND	ND	ND	-	-
Lactate (%)	3.28^a^	2.93^b^	1.41^d^	2.28^c^	0.158	0.030
Acetate (%)	1.15^b^	0.58^c^	2.54^a^	0.87^bc^	0.182	0.007
Lactate:acetate ratio	2.85^ab^	5.05^a^	0.56^b^	2.62^ab^	0.181	<0.001
Ensiled for 100 days						
pH	4.20	4.06	4.23	4.01	0.089	0.078
Ammonia-N (%)	0.39	0.40	0.35	0.37	0.032	0.095
Lactate (%)	2.44^ab^	2.73^a^	1.55^b^	2.82^a^	0.506	0.009
Acetate (%)	0.76^b^	0.47^b^	1.66^a^	0.81^b^	0.352	0.005
Lactate:acetate ratio	3.21^b^	5.81^a^	0.93^c^	3.48^b^	0.238	0.001

SEM, standard error of the mean; ND, not detected.

1) CON, silage with no inoculant; LP, silage inoculated with *L. plantarum* at 1.2×10^5^ cfu/g of forage; LB, silage inoculated with *L. buchneri* at 1.2×10^5^ cfu/g of forage; MIX, silage inoculated with mixture of LP and LB at 1:1 ratio.

a–c Means in the same row with different superscripts differ significantly (p<0.05).

**Table 3:** Effects of homo and hetero fermentative inoculants on chemical compositions and *in vitro* digestibility of sweet potato vine mixed with Italian ryegrass hay ensiled for 100 days (%, dry matter)

Item	Treatment1)				SEM	p value

	CON	LP	LB	MIX		
Dry matter	37.5	37.4	38.6	38.6	1.163	0.326
Crude protein	7.79	7.76	7.89	7.74	0.228	0.149
Ether extract	2.16	2.31	2.28	2.20	0.110	0.258
Crude ash	7.43	7.43	7.54	7.50	0.202	0.726
Neutral detergent fiber	62.0	61.5	62.3	62.6	0.919	0.414
Acid detergent fiber	40.7	40.5	41.0	40.6	0.936	0.886
Hemicellulose	21.2	21.0	21.3	22.0	0.968	0.514
IVD_DM_	58.9	58.5	58.0	59.5	0.595	0.420
IVD_NDF_	41.4	40.5	41.4	42.1	0.499	0.365

SEM, standard error of the mean; IVD_DM_, in vitro dry matter digestibility; IVD_NDF_, *in vitro* neutral detergent fiber digestibility.

1) CON, silage with no inoculant; LP, silage inoculated with *L. plantarum* at 1.2×10^5^ cfu/g of forage; LB, silage inoculated with *L. buchneri* at 1.2×10^5^ cfu/g of forage; MIX, silage inoculated with mixture of LP and LB at 1:1 ratio.

**Table 4:** Effects of homo and hetero fermentative inoculants on aerobic stability and microbial levels of sweet potato vine mixed with ryegrass hay ensiled for 100 days

Item	Treatment1)				SEM	p value

	CON	LP	LB	MIX		
Aerobic stability (h)	39.9^c^	36.4^c^	149.2^a^	60.1^b^	4.209	<0.001
LAB (log 10 cfu/g)	6.34^ab^	6.04^b^	6.20^b^	6.60^a^	0.189	0.008
Yeast (log10 cfu/g)	6.36	6.06	6.00	5.88	0.24	0.085
Mold (log 10 cfu/g)	4.62^ab^	5.50^a^	4.09^b^	4.37^ab^	1.252	0.018

SEM, standard error of the mean; LAB, lactic acid bacteria.

1) CON, silage with no inoculant; LP, silage inoculated with *L. plantarum* at 1.2×10^5^ cfu/g of forage; LB, silage inoculated with *L. buchneri* at 1.2×10^5^ cfu/g of forage; MIX, silage inoculated with mixture of LP and LB at 1:1 ratio.

a,b Means in the same row with different superscripts differ significantly (p<0.05).
